# Case-by-case combination of the prostate imaging reporting and data system version 2.1 with the Likert score to reduce the false-positives of prostate MRI: a proof-of-concept study

**DOI:** 10.1007/s00261-024-04506-2

**Published:** 2024-07-30

**Authors:** Rossano Girometti, Valeria Peruzzi, Paolo Polizzi, Maria De Martino, Lorenzo Cereser, Letizia Casarotto, Stefano Pizzolitto, Miriam Isola, Alessandro Crestani, Gianluca Giannarini, Chiara Zuiani

**Affiliations:** 1 Institute of Radiology, Department of Medicine (DMED), University of Udine, University Hospital S. Maria della Misericordia – Azienda Sanitaria-Universitaria Friuli Centrale (ASU FC), p.le S. Maria della Misericordia, 15 – 33100 Udine, Italy; 2 Division of Medical Statistics, Department of Medicine (DMED), University of Udine, pl.le Kolbe, 4 – 33100 Udine, Italy; 3 Pathology Unit, University Hospital S. Maria della Misericordia – Azienda Sanitaria-Universitaria Friuli Centrale (ASU FC), p.le S. Maria della Misericordia, 15 – 33100 Udine, Italy; 4 Urology Unit, University Hospital S. Maria della Misericordia – Azienda Sanitaria-Universitaria Friuli Centrale (ASU FC), p.le S. Maria della Misericordia, 15 – 33100 Udine, Italy; 5 Present Address: UOC Radiologia, Ospedale Civile SS. Giovanni e Paolo, ULSS 3 Serenissima, 6776 - 30122 Castello, Venezia, Italy

**Keywords:** Prostatic neoplasms, Multiparametric magnetic resonance imaging, Biopsy, PI-RADS

## Abstract

**Objectives:**

To retrospectively investigate whether a case-by-case combination of the Prostate Imaging Reporting and Data System version 2.1 (PI-RADS) with the Likert score improves the diagnostic performance of mpMRI for clinically significant prostate cancer (csPCa), especially by reducing false-positives.

**Methods:**

One hundred men received mpMRI between January 2020 and April 2021, followed by prostate biopsy. Reader 1 (R1) and reader 2 (R2) (experience of > 3000 and < 200 mpMRI readings) independently reviewed mpMRIs with the PI-RADS version 2.1. After unveiling clinical information, they were free to add (or not) a Likert score to upgrade or downgrade or reinforce the level of suspicion of the PI-RADS category attributed to the index lesion or, rather, identify a new index lesion. We calculated sensitivity, specificity, and predictive values of R1/R2 in detecting csPCa when biopsying PI-RADS ≥ 3 index-lesions (strategy 1) versus PI-RADS ≥ 3 or Likert ≥ 3 index-lesions (strategy 2), with decision curve analysis to assess the net benefit. In strategy 2, the Likert score was considered dominant in determining biopsy decisions.

**Results:**

csPCa prevalence was 38%. R1/R2 used combined PI-RADS and Likert categorization in 28%/18% of examinations relying mainly on clinical features such as prostate specific antigen level and digital rectal examination than imaging findings. The specificity/positive predictive values were 66.1/63.1% for R1 (95%CI 52.9–77.6/54.5–70.9) and 50.0/51.6% (95%CI 37.0-63.0/35.5-72.4%) for R2 in the case of PI-RADS-based readings, and 74.2/69.2% for R1 (95%CI 61.5–84.5/59.4–77.5%) and 56.6/54.2% (95%CI 43.3-69.0/37.1-76.6%) for R2 in the case of combined PI-RADS/Likert readings. Sensitivity/negative predictive values were unaffected. Strategy 2 achieved greater net benefit as a trigger of biopsy for R1 only.

**Conclusion:**

Case-by-case combination of the PI-RADS version 2.1 with Likert score translated into a mild but measurable impact in reducing the false-positives of PI-RADS categorization, though greater net benefit in reducing unnecessary biopsies was found in the experienced reader only.

**Graphical Abstract:**

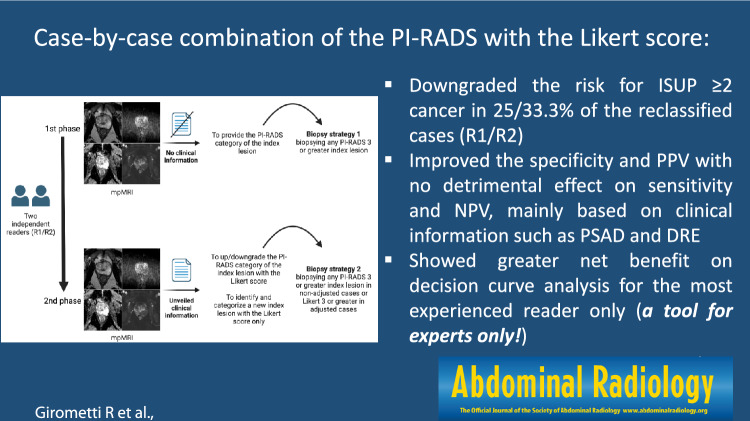

**Supplementary Information:**

The online version contains supplementary material available at 10.1007/s00261-024-04506-2.

## Introduction

Since its introduction in 2012 [1] and revision as version 2 in 2015 [2], the Prostate imaging reporting and data system (PI-RADS) has become the most widely accepted standard for interpreting multiparametric magnetic resonance imaging (mpMRI) of the prostate. Current version 2.1 [3], released in 2019, has been validated by several studies [4–8] and, according to a recent metanalysis, shows pooled positive predictive value (PPV) for clinically significant prostate cancer (csPCa) of 16, 59 and 85% for PI-RADS 3, 4 and 5 category, respectively [9]. Though the PI-RADS promotes a standardized lesion-based scoring approach, interpretation remains subjective in several instances, thus explaining its moderate inter-reader agreement only with version 2 and 2.1 [10, 11]. Current limitations of version 2.1 also include the need to clarify some interpretation criteria, lack of definite criteria for scoring the central zone, lack of assessment of the prostate background potentially affecting cancer detection [12] and, importantly, still limited specificity translating in too many unnecessary biopsies [7].

Not surprisingly, the PI-RADS is not of universal use in the setting of initial diagnosis of csPCa. While the joint societies' European guidelines endorse it with a "strong" strength rating [13], other recommendations favor the Likert score as the preferred alternative for reporting prostate MRI [14, 15]. Comparably to the PI-RADS, the Likert score expresses the risk that a mpMRI observation is a csPCa on an ascending 1–5 scale, though this system works as a gestalt subjective assessment not relying on a dominant sequence or specific criteria to define each risk category [16]. This allows for much flexibility when interpreting findings that are difficult-to-categorize with the PI-RADS, and the possibility to take clinical information into account, e.g., age, prostate-specific antigen (PSA) level, PSA density (PSAD), family history, and so on [16, 17].

A few studies comparing both systems on an intra-patient basis found that the Likert score has the potential for greater diagnostic accuracy [18] and improved specificity compared to PI-RADS version 2 [19]. This suggests the potential for maximizing cancer detection while avoiding unnecessary biopsies, which still represent the Achilles's heel of prostate mpMRI [20]. On the other hand, the absence of standardized rules of image interpretation translates into its dependence on the radiologist's experience [16, 18] and limited potential for reproducibility across different institutions and practice settings compared to the relatively objective PI-RADS [16]. A recent British audit of cancer yields after prostate MRI found PI-RADS version 2 and the Likert score clinically equivalent, with most discrepancies confined to the PI-RADS 4 category [21]. Given the difficulty in establishing the superiority of one system over the other, we hypothesized that a two-step combined use of both systems could maximize the related advantages while minimizing disadvantages, thus potentially improving the diagnostic performance, especially in terms of reducing false-positive cases. We assumed that while the PI-RADS version 2.1 can represent the basis for reporting (first step), the radiologist could refine lesion categorization with the Likert score in all those selected cases in which the PI-RADS is perceived as not fully catching the complexity of risk assessment (second step).

This study aimed to assess whether the above-mentioned case-by-case strategy of combining the PI-RADS version 2.1 categories with the Likert score reduces the number of false-positive cases for csPCa and the appropriateness of mpMRI-informed biopsy decisions.

## Material and methods

### Study population and standard of reference

The Institutional Review Board approved this monocentric study. The acquisition of informed consent was waived because of the retrospective design.

**Fig. 1:**
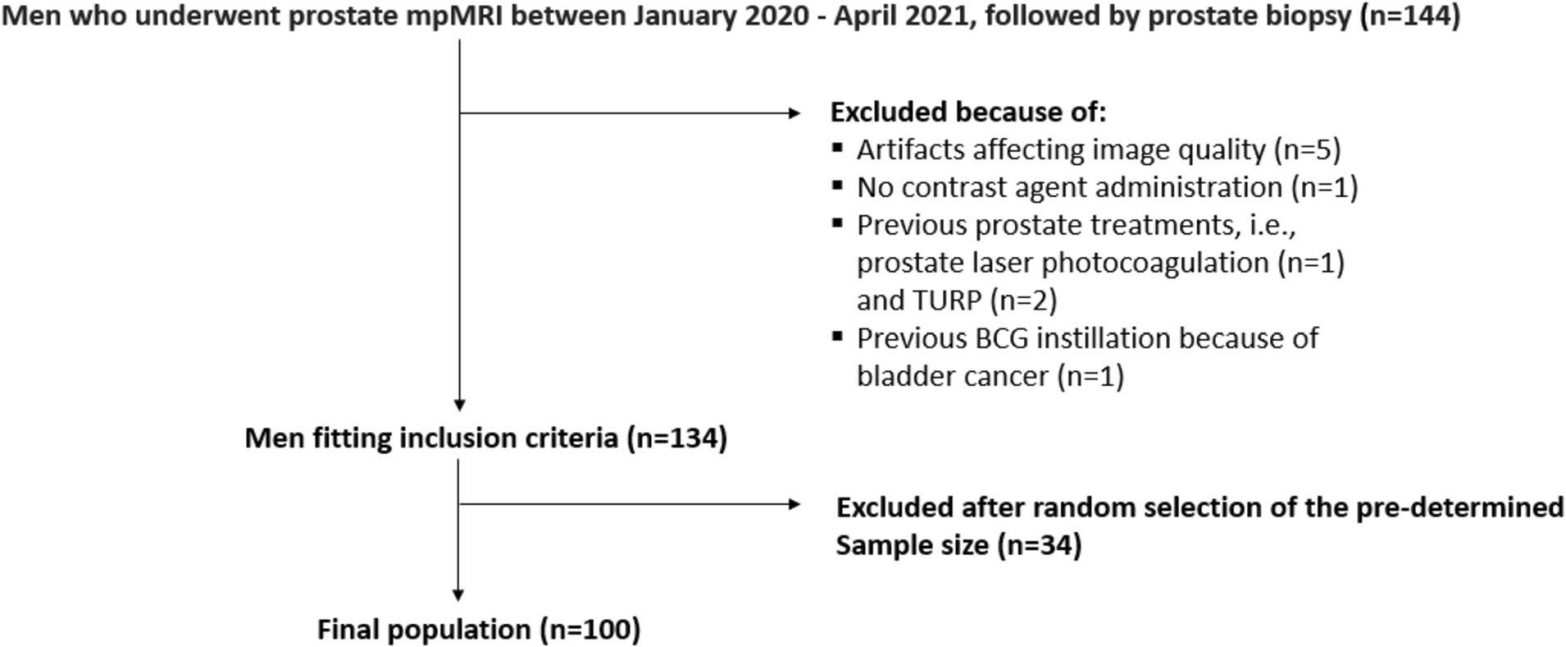
Study flowchart. BCG = bacillus Calmette-Guérin; mpMRI = multiparametric magnetic resonance imaging; TURP = transurethral resection of the prostate

We searched the institutional database for all consecutive ≥ 18-year-old men who underwent prostate mpMRI followed by prostate biopsy between January 2020 and April 2021. Indications to mpMRI were clinical suspicion of csPCa (PSA value ≥ 3 ng/ml and/or positive digital rectal examination) in biopsy-naïve men or persistent clinical suspicion of csPCa despite one or more prior negative prostate biopsies. We identified144 eligible subjects who received prostate through the transperineal route by one of three urologists using software-assisted mpMRI-ultrasound guidance (Applio 300, Toshiba/Canon). The biopsy included 4 target cores (2 in-target and 2 peri-target) on PI-RADS ≥ 3 lesions, followed by 12 systematic cores. Per internal policy, patients with PI-RADS 1–2 examinations and high clinical risk received only systematic biopsy. After excluding 10 men because of the exclusion criteria shown in Fig. 1, we used freely available software (https://www.randomizer.org/) to randomly select 100 over the remaining 134 men as the final study population (Fig. 1). This number of patients was defined in advance when planning the study as a balance between the available time for performing the readings and the study duration. All included men were Caucasian.

The standard of reference was represented by ISUP-compliant histological examination performed on biopsy cores [22] by one of three genitourinary pathologists (5–30 years of experience). csPCa was defined as a lesion showing the highest ISUP grading group ≥ 2 on systematic or targeted biopsy.

### Imaging protocol

Examinations were acquired on a 1.5 T (MAGNETOM Aera, Siemens Healthineers) or a 3.0 T MRI equipment (Achieva, Philips Medical Systems) in 13/100 and 87/100 cases, respectively. A 32-channel surface coil was used. All patients received preliminary cleansing enema and i.m. administration of 20 mg hyoscine butylbromide (Buscopan, Boehringer Ingelheim) as an antiperistaltic agent.

Acquisition parameters are detailed in Supplementary Tables 1 and 2. On the 1.5 T magnet, the maximum b-value in the second diffusion-weighted sequences was interpolated up to 1400 s/mm^2^. The apparent diffusion coefficient (ADC) map was built upon a monoexponential fitting of signal decay versus b-values of the first diffusion-weighted sequence (maximum b = 1000 s/mm^2^). Dynamic contrast-enhanced imaging (DCE) was acquired intravenously after administering 0.2 mL/Kg of gadoteridol (Prohance, Bracco) at an injection rate of 3 ml/s using a remote-controlled power injector (Medrad Spectris Solaris EP). DCE series was presented as native images and subtracted ones.

### Image analysis

Two readers independently analyzed images on a Picture Archiving and Communication System console (Suite Estensa, Ebit). Readers included one radiologist (R1) with an experience of > 3000 examinations (R.G.) and a non-experienced radiologist resident (R2) mentored by R1 during clinical activity (< 200 readings) (V.P.). A study coordinator (P.P.) showed them mpMRI examinations using a two-phase strategy.

In the first phase, blinded to clinical information, readers were allowed to report up to four lesions to be scored with the PI-RADS version 2.1 (“PI-RADS” from here on out) [3] and asked to clearly identify the index lesion as the one showing the highest PI-RADS category or the largest size in the case of more lesions with the same PI-RADS category. When readers found no lesions, the examination was assumed to include a PI-RADS 1 "index lesion" for analysis. In the second phase, the coordinator disclosed clinical data, including age, results from prior biopsy, if any, last PSA value, results of the digital rectal examination, prostate volume calculated in the original mpMRI report, PSAD, ongoing therapy with alpha-blockers if any, family history of csPCa, and symptoms if any. Based on those clinical features and depending on the mpMRI appearance, readers were then allowed to make case-by-case additional use of the Likert score according to the following rules: (i) combining the PI-RADS category of the lesions with a Likert score, e.g., to reinforce a level of suspicion (e.g., PI-RADS 3 combined with Likert 3 score) or instead upgrading or downgrading it (e.g., PI-RADS 3 combined with Likert 2 or PI-RADS 3 combined with Likert 4); (ii) identifying and categorize a new lesion and assign it a Likert score only. Using the Likert score was not mandatory, so readers were asked to explain reasons for doing so on a case-by-case basis, detailing the number and type of clinical variables and imaging findings that triggered combined scoring. Imaging features supporting Likert scoring were those summarized by Latifoltojar et al. in Supplementary Tables 5, 6 and 7 of their paper [17], as well as the PI-RADS descriptors for T2-weighted imaging, DWI and DCE [3]. Differently from the PI-RADS, we did not establish in advance which imaging or clinical feature should have been selected or privileged for image analysis, nor defined exact combinatory rules to achieve a certain Likert score. Readers were also free to integrate clinical information with no predefinite rules, except establishing that the PSAD value to be considered “suspicious” was 0.15 ng/mL/mL (not a standalone criterion for malignancy). Our strategy aimed at: (a) reflecting the subjective nature of the Likert system and facilitate the comparison with previous works on the same topic; (b) to prevent the risk of testing a set of combinatory rules rather than the properly said Likert score; (c) to prevent the risk that a set of definite combinatory rules could overinflate the performance of the less experienced reader.

The Likert score was assumed to express the risk that a mpMRI finding was a csPCa as follows: 1 = highly unlikely; 2 = unlikely; 3 = equivocal; 4 = likely; 5 = highly likely [21].

### Statistical analysis

After observing non-normal data distribution with the Shapiro–Wilk test, we used the median and the interquartile range (IQR) to report continuous variables. Relevant proportions were coupled with 95% confidence intervals (95% CI). Descriptive statistics was also used to report how many lesions were found by R1 and R2 and how they were categorized with the PI-RADS and Likert scores.

Concerning PI-RADS categorization, we decided not to run an inter-reader agreement analysis because readers could detect different lesions. We then calculated the per-category rate of concordant categorizations, i.e., how many times R1 and R2 assessed the same index lesion as PI-RADS 1–2, PI-RADS 3, or PI-RADS 4–5 over the total number of index lesions scored with the same PI-RADS category.

Based on the rules of comparison between mpMRI results and prostate biopsy shown in Table 1, we calculated the per-index lesion sensitivity, specificity, PPV and negative predictive value (NPV) for csPCa of two different biopsy strategies, as follows: (i) strategy 1 (PI-RADS categorization only), i.e., biopsying any index lesion categorized PI-RADS ≥ 3; (ii) strategy 2 (PI-RADS categorization combined with the Likert score), i.e., biopsying any index lesion categorized as PI-RADS ≥ 3 (in cases receiving PI-RADS categorization only) or Likert ≥ 3 (in cases receiving combined scoring). In strategy 2, the Likert categorization, when attributed, was considered dominant compared to the PI-RADS. E.g., a PI-RADS 2 lesion upgraded to Likert 4 was assumed to be biopsied, while a PI-RADS 3 lesion downgraded to Likert 2 was assumed to avoid biopsy. For analysis, newly identified lesions in reading phase 2 showing a Likert score greater than the PI-RADS category of the index lesion established in reading phase 1 were assumed to represent the index lesion for biopsy strategy 2.

**Table 1:** Summary of the criteria used to categorize index lesions as true positive, false positive, false negative, and true negative according to the prostate biopsy result

	Positive biopsy (At least one core containing ISUP ≥ 2 cancer)	Negative biopsy (No cores containing ISUP ≥ 2 cancer)
Positive mpMRI		
Strategy 1: index lesion categorized PI-RADS ≥ 3 Strategy 2: index lesion categorized PI-RADS ≥ 3 (PI-RADS categorization only) or Likert ≥ 3 (combined PI-RADS Likert categorization)	True positive Positive target biopsy and/or a positive systematic biopsy in at least one adjacent prostate quadrant	False positive Negative target biopsy and negative systematic biopsy in all the adjacent prostate quadrants
Negative mpMRI		
Strategy 1: index lesion categorized PI-RADS ≤ 2 Strategy 2: index lesion categorized PI-RADS ≤ 2 (PI-RADS categorization only) or Likert ≤ 2 (combined PI-RADS Likert categorization)	False negative Case 1 (clearly defined index lesion): positive target biopsy (if any) and/or a positive systematic biopsy in at least one adjacent prostate quadrant Case 2 (PI-RADS 1 examination with no clearly defined index lesion): positive systematic biopsy	True negative Case 1 (clearly defined index lesion): negative target biopsy (if any) and/or negative systematic biopsy in all the adjacent prostate quadrants Case 2 (PI-RADS 1 examination with no clearly defined index lesion): negative systematic biopsy

"Quadrant(s)" refers to the sectorial map of the Prostate Imaging Reporting and Data System (PI-RADS) version 2.1, while "adjacent" refers to any quadrant neighboring the index lesion on either the same prostate level (base, mid-gland, apex) or any same quadrant in the adjacent prostate level

*ISUP* International Society of Urogenital Pathology grading group,* mpMRI* multiparametric magnetic resonance imaging

The clinical impact of both biopsy strategies was assessed with the decision curve analysis [23], assuming that the reference "treat all" and "treat none" strategies meant to biopsy all men and biopsy none, respectively. Net benefit, i.e., the balance between the advantage of diagnosing true positives weighted for the harm of biopsying false positives, was calculated at disease threshold probabilities of 10, 15, 20, 25, and 30%, respectively.

Calculations were performed using commercially available software (MedCalc software bv, version 18.11.16), except for decision analysis, which was run on Stata using source codes freely available at https://www.mskcc.org/departments/epidemiology-biostatistics/biostatistics/decision-curve-analysis.

## Results

### Study population

The median age of the men included was 66.0 years (IQR 61.0–72.0). The median serum PSA and PSAD were 6.44 ng/mL (IQR 4.85–8.94) and 0.11 ng/mL/mL (IQR 0.07–0.17), respectively. Seventy-nine/100 men were biopsy-naïve, while the remaining 21/100 showed previous negative biopsy. csPCa was found in 38/100 men (38%; 95% CI 29.59–46.41). Lesions included 17/38 ISUP 2 cancers (44.73%), 12/38 ISUP 3 cancers (31.57%), 7/38 ISUP 4 cancers (18.42%), and 2/38 ISUP 5 cancers (5.26%). Clinically insignificant cancer (ISUP 1) was found in 12/100 men (12%).

### PI-RADS categorization

R1 and R2 reported 119 and 131 mpMRI findings on one hundred men, respectively. Table 2 summarizes the distribution of their PI-RADS categories. Index lesions were found in the peripheral zone and transition zone in 55/100 and 20/100 cases by R1 and 68/100 and 18/100 cases by R2, respectively. The remaining 25/100 cases (R1) and 14/100 cases (R2) were PI-RADS 1 "index lesions" not corresponding to definite mpMRI observations.

**Table 2:** Distribution of the PI-RADS version 2.1 assignments made by reader 1 and reader 2

		PI-RADS version 2.1 category number (%; 95%CI)					Total number of lesions
		1	2	3	4	5	
Reader 1	All lesions	25 (21.01%; 14.08–29.43)	23 (19.33%; 12.66–27.58)	12 (10.1%; 5.32–16.95)	41 (34.45%; 25.98–43.72)	18 (15.13%; 9.22–22.85)	119
	Index lesions	25 (25%; 16.88.34.66)	18 (18%; 11.03–26.95)	9 (9%; 4.20–16.40)	30 (30%; 21.24–39.98)	18 (18%; 11.03–26.95)	100
Reader 2	All lesions	14 (10.69%; 5.97–17.28)	26 (19.85%; 13.39–27.71)	22 (16.8%; 10.83–24.31)	54 (41.22%; 32.70–50.15)	15 (11.45%; 6.55–18.18)	131
	Index lesions	14 (14%; 7.87–22.37)	19 (19%; 11.84–28.07)	14 (14%; 7.87–22.37)	40 (40%; 30.33–50.28)	13 (13%; 7.11–21.20)	100

Readers identified the same index lesion in 66/100 cases (66%; 95% CI 50.40–69.60), providing the same PI-RADS categorization in 55/66 cases (83.3%; 95% CI 74.34–92.32). In particular, the rate of concordant categorizations was 15/55 (27.3%; 95% CI 15.50–39.04) for PI-RADS 1–2 assignments, 1/55 (1.8%; 95% CI 00.05–05.35) for PI-RADS 3 assignments, and 39/55 (70.9%; 95% CI 58.91–82.91) for PI-RADS 4–5 assignments. The eleven cases of discordant categorizations are detailed in Supplementary Table 3.

### Combined PI-RADS-Likert score categorization

**Fig. 2:**
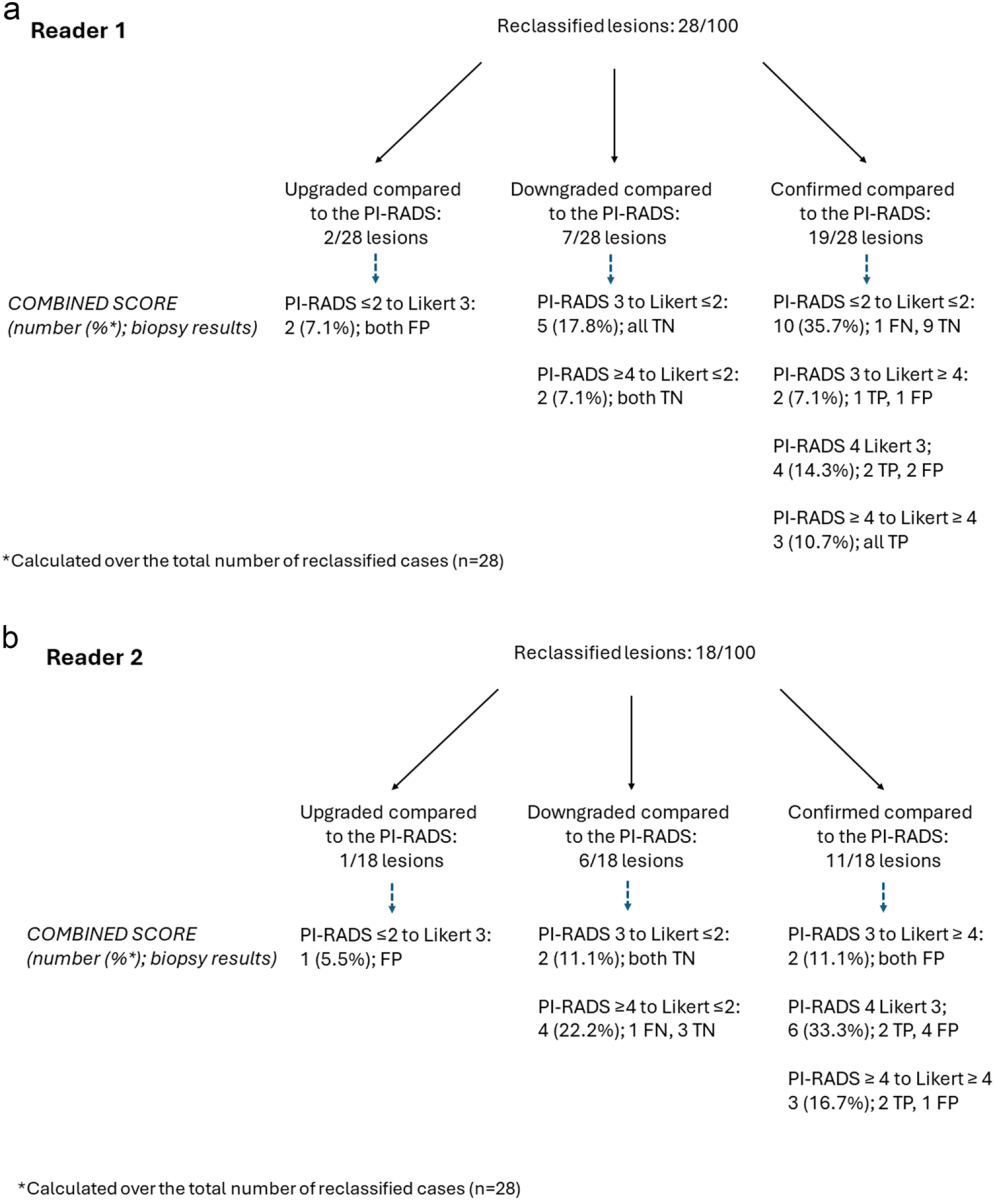
Distribution of cases in which reader 1 (**a**) and reader 2 (**b**) used the Likert score to complement the PI-RADS categorization of index lesions. FN = false-negative; FP = false-positive; PI-RADS = Prostate Imaging Reporting and data System version 2.1; TN = true negative; TP = true-positive

R1 and R2 provided combined PI-RADS-Likert categorization of the index lesion in 28/100 (28%; 95% CI 19.20–36.80) and 18/100 (18%; 95% CI 10.47–25.53) cases, respectively, as summarized in Fig. 2 and in Supplementary Table 4. The latter shows that, for both readers, the use of the Likert score was mostly supported by PSAD values and the results of DRE.

**Fig. 3:**
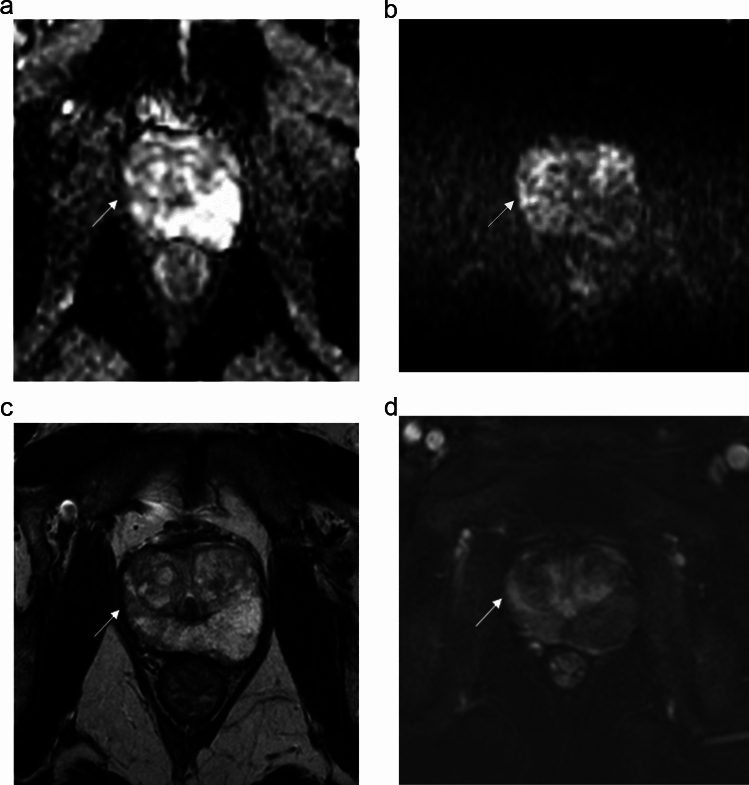
Case of Likert scoring by reader 1 reinforcing the level of suspicion of PI-RADS categorization in a 53-year-old man with a prostate-specific antigen level density of 0.08 ng/mL/mL and negative digital rectal examination. The index lesion in the right anterior peripheral zone of the midgland showed wedge-shaped mild hypointensity on the apparent diffusion coefficient map (arrow in **a**) and wedge-shaped mild hyperintensity on b = 2000s/mm^2^ image (**b**), slight hypointensity on T2-weighted imaging (arrow in **c**) and early focal enhancement after contrast administration (arrow in **d**). The lesion was assessed as PI-RADS 2 and Likert 2. Transperineal systematic biopsy cores in the same quadrant and adjacent quadrant showed gland atrophy/subatrophy and chronic prostatitis

R1 assigned a Likert ≤ 2 score to PI-RADS ≤ 2 findings in 10/28 cases (35.7%) and a Likert ≥ 3 score to PI-RADS ≥ 3 findings in 9/28 cases (32.1%), suggesting that Likert scoring was used to reinforce the lesion risk in around two-thirds of cases (Fig. 3). The same trend was observed for R2, who assigned a Likert ≥ 3 score to PI-RADS ≥ 3 findings in 11/18 cases (61.1%). Most reinforcements of suspicious cases regarded PI-RADS 4 assignments (7/9 for R1 and 9/11 for R2). R1 observed no PI-RADS 5 or Likert 5 cases, while R2 upgraded 3 PI-RADS 4 lesions to Likert 5.

**Fig. 4:**
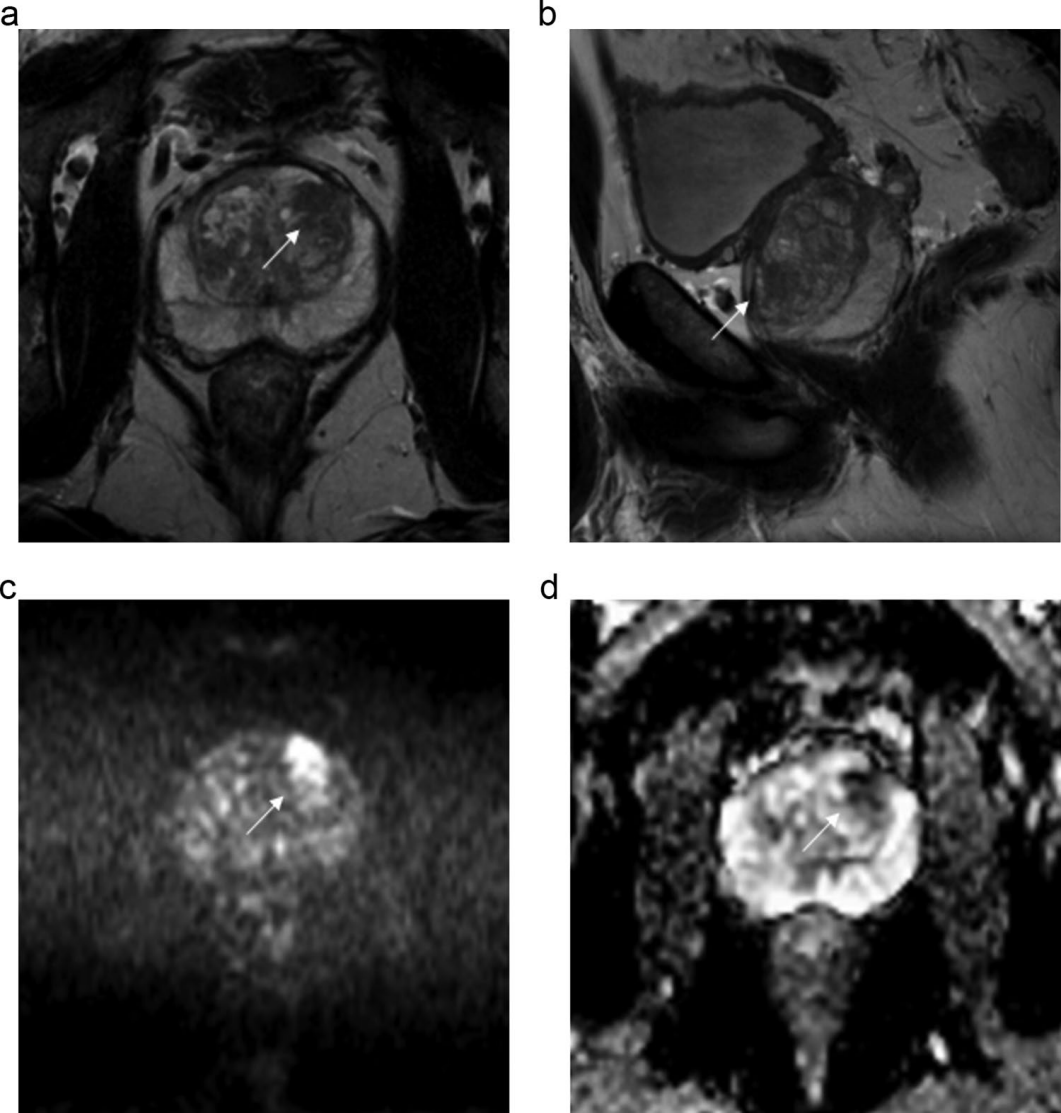
Case of Likert-induced downgranding of lesion suspicion by reader 1 in a 62-year-old man. A mildly-hypointense atypical nodule in the left anterior transition zone of the midgland (arrow in **a** and **b**) showed restricted diffusion with marked hyperintensity on b = 2000s/mm^2^ image (**c**) and marked hypointensity on the apparent diffusion coefficient map (**d**), and was categorized as a PI-RADS 2 upgraded to 3. Based on prostate-specific antigen level density of 0.07 ng/mL/mL and negative digital rectal examination, reader 1 downgraded the level of suspicion to Likert 2. A targeted prostate biopsy showed chronic prostatitis

The secondary main trend consisted in assigning a Likert ≤ 2 score to PI-RADS ≥ 3 findings, i.e., 7/28 (25.0%) cases by R1 and 6/18 (33.3%) cases by R2, respectively. Reclassification beneath the threshold for biopsy translated into a switch from false-positives to true-negatives in all cases (Fig. 4). In a minority of cases, R1 and R2 assigned a Likert ≥ 3 score to PI-RADS ≤ 2 lesions (2/28 and 1/18 cases, respectively), all of which were found to be false-positives at systematic biopsy. Reclassifications are shown in Fig. 2. As an overall balance between the false-positive cases saved or induced by the use of the Likert score, strategy 2 could have avoided 5 and 4 unnecessary biopsies for R1 and R2, respectively.

### Diagnostic performance

The diagnostic performance of biopsy strategies 1 and 2 is shown in Table 3. For both readers, strategy 2 translated into increased specificity and PPV while maintaining comparable sensitivity and NPV.

**Table 3:** Diagnostic performance of biopsy strategy 1 and biopsy strategy 2 (see the main text for definitions)

	Biopsy strategy 1					Biopsy strategy 2				
	Sn % (95%CI)	Sp % (95%CI)	PPV % (95%CI)	NPV % (95%CI)	TP/FP/TN/FN (number)	Sn % (95%CI)	Sp % (95%CI)	PPV % (95%CI)	NPV % (95%CI)	TP/FP/TN/FN (number)
R1	94.7 (82.25–99.3)	66.1 (52.99–77.66)	63.1 (54.56–70.99)	95.3 (84.0–98.76)	36/21/41/2	94.7 (82.2–99.3)	74.2 (61.5- 84.5)	69.2 (59.4–77.5)	95.8 (85.5–98.8)	36/16/46/2
R2	86.8 (71.9–95.6)	50.0 (37.0–63.0)	51.6 (35.5–72.4)	86.1 (58.5–122.2)	33/31/31/5	84.2 (68.7–94.1)	56.5 (43.3–69.0)	54.2 (37.1–76.6)	85.4 (59.5–119.7)	32/27/35/6

*Sn* sensitivity, *Sp* specificity, *PPV* positive predictive value, *NPV* negative predictive value, *TP* true positive, *FP* false positive, *TN* true negative, *FN* false negative, *R1* reader 1, *R2* reader 2

**Fig. 5:**
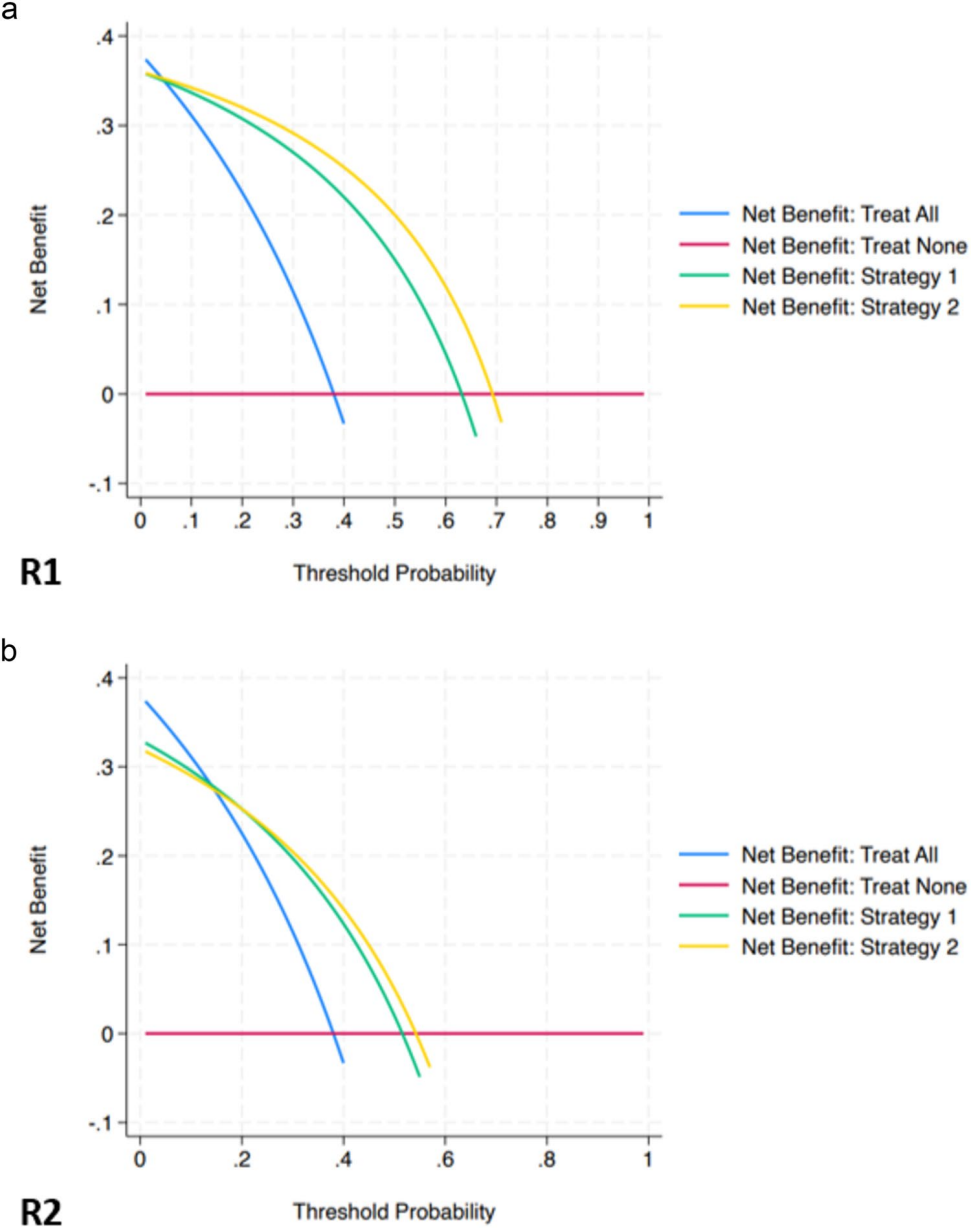
Decision curve analysis for reader 1 (**a**) and reader 2 (**b**) (see the main text for details)

Concerning the clinical impact for R1, decision curve analysis (Fig. 5) showed greater net benefit of strategy 2 compared to strategy 1 over the whole range of disease probability, with net benefit values at 10, 15, 20, 25 and 30% of csPCa likelihood of 0.34 versus 0.33, 0.33 versus 0.32, 0.32 versus 0.30, 0.30 versus 0.29 and 0.29 versus 0.27, respectively. In the case of R2, the curves of strategy 2 and strategy 1 largely overlapped up to around 25% threshold probability, with comparable net benefit values at 10% (0.29), 15% (0.27), and 20% of csPCa likelihood, and greater net benefit values at 25% (0.23 versus 0.22) and 30% (0.20 vs. 0.19) disease probability.

## Discussion

In this study, we observed that, when combining the PI-RADS version 2.1 categorization of prostate index lesions with a case-by-case use of the Likert score, R1 and R2 downgraded the risk of csPCa beneath the threshold actioning prostate biopsy in 25 and 33.3% of the reclassified cases, respectively. This translated into increased mpMRI specificity and PPV in diagnosing ISUP ≥ 2 prostate cancer, with no detrimental effect on sensitivity and NPV. While this trend was observed in both readers, the net benefit on decision curve analysis improved for R1 only, supporting previous observations that adequate reader experience is the prerequisite for using the Likert scale [24, 25]. This is related to the fact that most experienced readers are able to account for additional clinical and imaging factors when interpreting mpMRI and, in turn, make image interpretation more flexible and nuanced. Of note, greater net benefit was observed across the whole range of csPCa probability in our population (79% biopsy-naïve men and 21% re-biopsy patients).

As far as we know, previous studies did not investigate a similar strategy but rather compared the PI-RADS (version 1 or 2) versus the Likert score as alternative systems for diagnosis [18, 19, 26–29]. In line with our results, one of those works by Zawaideh et al. on 199 men [19] found that, being equal the sensitivity (94%) and NPV (96%), the use of the Likert score translated into lower positive call rate, and in turn greater per-lesion specificity and PPV for ISUP ≥ 2 cancers than the PI-RADS version 2 (77 versus 66% and 66 versus 58%, respectively). Differently from Khoo et al. [18], we did not observe an increase in cancer detection rate since sensitivity remained stable for R1 (94.7%) or minimally dropped for R2 (from 86.8 to 84.2%). This is in line with the fact that the Likert score upgraded the PI-RADS risk in a very minority of cases, and most times inappropriately, e.g., 2/2 cases upgraded from PI-RADS ≤ 2 to Likert 3 and 1/2 cases upgraded from PI-RADS 3 to Likert ≥ 4 were false-positive for the most experienced reader. Our results suggest that the Likert-induced upgrading is expectedly rare and should be regarded with caution as a trigger for biopsy, though further studies should confirm this issue and assess how to overcome it.

Our findings are more directly comparable with those by Stevens et al. [30], who performed logistic regression to identify Likert findings predicting csPCa and, in turn, built a model to automatically adjust indeterminate PI-RADS 3 cases (version 2) with the Likert score. In the testing cohort, the adjustment translated into an increase in specificity from 30.3 to 74.9%, comparable to the one we observed for our most experienced reader when using strategy 2 (74.2%). However, we did not focus analysis on indeterminate cases only and showed lower PI-RADS 3 call rates (10.1% for R1 and 16.8% for R2) than those Authors (135/411 men, i.e., 32.8% in the building cohort, and 159/380 men, i.e., 41.8% in the testing cohort). One can assume that, even though the Authors' model determined a comparable increase in specificity and PPV in a validation study [26], our results are at lower risk of overestimation in favor of Likert-induced effects over the entire spectrum of PI-RADS categories.

A strength of our study is that we blinded readers to clinical information when reporting with the PI-RADS, thus eliminating those confounders that could have translated into using "modified PI-RADS categories" close to Likert ones in clinical practice and previous trials [26]. Using this strategy translated into accurate lesion risk reduction, and, in turn, false-positives, e.g., as occurred for R1 in seven PI-RADS ≥ 3 cases reclassified as Likert ≤ 2 which were found to be inflammation on prostate biopsy (Suppl. Table 4). Though in a different setting, our results compare to those by Zawaideh et al. [19], who observed significantly more Likert negative/PI-RADS positive than Likert positive/PI-RADS negative cases. Notably, both readers used the Likert score mainly to reinforce the level of suspicion already expressed by the PI-RADS category, in line with the fact that this system expands image analysis and risk stratification from the lesion-based level of the PI-RADS to a more comprehensive patient-level. In the case of R1, this occurred mostly to reinforce a PI-RADS ≤ 2 category as a Likert ≤ 2 score (10/28 reclassified cases), in accordance with a recent audit of cancer yield showing that negative mpMRIs are a major source of agreement between PI-RADS version 2 and Likert scoring [21]. One can hypothesize that selective reporting of both the PI-RADS and Likert score could help identifying those cases in clinical practice and research in which clinical information was determinant in shaping the above-mentioned “modified PI-RADS” categories. A more systematic assessment and quantification could be helpful to further refine PI-RADS categories and provide more nuanced risk stratification.

As this was a proof-of-concept study, we did not run a systematic analysis of how much reproducible a selective use of additional Likert scoring can be, and whether it can depend on lesion location (i.e., peripheral zone versus transition zone findings) or other factors, e.g., how much complete the available clinical information is at the time of mpMRI reporting. While the Likert score is not-standardized by definition, it was found to compare the diagnostic performance of the PI-RADS [21], effectively impact biopsy decisions in reference studies such as the MRI-FIRST [31], and potentially prompt which imaging features can be helpful in future revisions of the PI-RADS [21]. In our study, both readers relied more on clinical variables than imaging findings when using the Likert score (Supplementary Table 4), especially PSAD and DRE compared to the remaining clinical information available during reading phase 2 (age, prior biopsy, PSA, prostate volume, ongoing therapy with alpha-blockers, family history of csPCa, and symptoms). This result is in line with the role that PSAD and DRE have in shaping biopsy decisions and defining patient risk categories, respectively [13]. Our results support the concept that, while the most reproducible and impacting features supporting a selective use of the Likert score should be further elucidated, the strategy we investigated is of clinical added value in reducing the false positives in the real world. Further studies should also assess whether Likert-adjusted PI-RADS categories compare with risk calculators in assessing the pre-biopsy risk of harboring csPCa [32] or can represent additional variables to be included in clinical-imaging-based risk models, assumed that the same expert readers who provided reliable PI-RADS categorization can properly refine them as we showed.

We must acknowledge several study limitations. Given the retrospective design, we could not perform a targeted biopsy of index lesions found by Likert scoring only, so a systematic biopsy was used as a surrogate standard of reference. Second, we did not compare our approach to strategies proven to reduce the false-positive rate (e.g., adjusting the PI-RADS with PSAD [13]) or multivariable models stratifying patients’ risk by combining clinical features with mpMRI findings [32]. However, in the absence of a definite strategy on how to refine PI-RADS categories, the Likert adjustment strategy could be used in high-volume centers and multidisciplinary contexts where the urologist and other professionals can become familiar with the increased complexity of the mpMRI report, and more likely trust the Likert score as the "dominant" category for shaping biopsy decision (e.g., when a PI-RADS 4 lesion is downgraded to Likert 2). At the same time, this strategy could help less experienced readers to capitalize on the more standardized approach of the PI-RADS during the learning curve phase while having the capability, under supervision, to face more difficult cases with the flexibility inherent to the Likert score. Third, we only included biopsied patients, thus making difficult understanding how the combined PI-RADS-Likert categorization can work in low risk patients with negative mpMRI. Finally, R2 was a resident mentored by R1 in clinical practice, suggesting that her criteria for using the Likert score can largely reflect those those of R1, and, in turn, limit the generalizability of our inter-reader comparison outside the monocentric setting of this study. This could be further emphasized by the fact that the use of the PI-RADS version 2.1 translated into a diagnostic performance of R2 close to that of the experienced radiologist in our study, suggesting that the effect of combining the Likert score with a standardized system should be tested on a larger scale in less experienced readers.

In conclusion, our proof-of-concept study supports the hypothesis that combining the PI-RADS version 2.1 categories with the Likert score can improve the specificity and PPV of prostate mpMRI with no detrimental effect on sensitivity and NPV. Regardless of readers’ experience, clinical features (especially PSAD and DRE) were the most impactful ones in determining combined PI-RADS and Likert scoring. However, the reproducibility of the factors triggering the selective use of the Likert score should be tested on a larger scale. While overall mild, the improvement translated into greater net benefit in shaping biopsy decisions in the case of R1, suggesting that this strategy can be easily and effectively used by more experienced readers in clinical practice.

## Supplementary Information

Below is the link to the electronic supplementary material.

Supplementary file1 (PDF 164 KB)
