# Supplementary material for: Case-by-case combination of the prostate imaging reporting and data system version 2.1 with the Likert score to reduce the false-positives of prostate MRI: a proof-of-concept study
Source: Abdom Radiol (NY). 2024 Jul 30;49(12):4273–85. doi: 10.1007/s00261-024-04506-2 (PMC11522071; doi:10.1007/s00261-024-04506-2)
Supplement: Supplementary file 1 — Supplementary file1 (PDF 164 KB) [file 261_2024_4506_MOESM1_ESM.pdf]

SUPPLEMENTARY MATERIAL

ABDOMINAL RADIOLOGY

Case-by-case combination of the Prostate Imaging reporting and Data System version 2.1 with the Likert score when interpreting prostate MRI: a proof-of-concept study.

Prof. Rossano Girometti, MD[1], Dr. Valeria Peruzzi, MD[1], Dr. Paolo Polizzi, MD[1(2)], Dr. Maria De Martino, MSc[3], Prof. Lorenzo Cereser, MD[1], Dr. Letizia Casarotto, MD[4], Dr. Stefano Pizzolitto, MD[4], Prof. Miriam Isola, MSc[3], Dr. Alessandro Crestani, MD[5], Dr. Gianluca Giannarini*, MD[5], Prof. Chiara Zuiani*, MD[1]

Corresponding Author: Prof. Rossano Girometti, MD

e-mail: rossano.girometti@uniud.it

Address: Institute of Radiology, Department of Medicine (DMED), University of Udine, University Hospital S. Maria della Misericordia – Azienda Sanitaria-Universitaria Friuli Centrale (ASU FC), p.le S. Maria della Misericordia, 15 – 33100 Udine, Italy

Supplementary Table 1 - Acquisition parameters for 1.5 Tesla magnetic resonance imaging (MRI) of the prostate. DCE = dynamic contrast-enhanced; DWI = diffusion-weighted imaging; FOV = field of view; SENSE = sensitivity encoding; SPAIR = spectral adiabatic inversion recovery; TE = time of echo; TR = time of repetition; TSE = turbo spin echo; SS-EPI = single-shot echo-planar imaging; THRIVE = T1-weighted high-resolution isotropic volume examination; EPI = echo planar imaging.

	**Double-sequence DWI**		**TSE T2-weighted imaging**	**DCE**
	**First sequence***	**Second sequence****		
**Sequence**	SS-EPI	SS-EPI	TSE	VIBE
**Weighting**	DWI	DWI	T2	T1
**Acquisition**	axial	axial	axial/coronal/sagittal (at least 2 planes)	axial
**TR (ms)**	4100	4100	2810/2810/2900	4.5
**TE (ms)**	64	68	105/105/113	1.66
**Echo train lenght**	-	-	8/16/16	-
**EPI factor**	122	122	-	-
**Half scan factor**	0.75	0.75	no	no
**FOV (mm x mm)**	200x200	200x200	200x200	280x280
**Acquisition voxel size (mm x mm x mm)**	1.6 x 1.5 x 3.5	1.6 x 1.6 x 3.5	0.7 x 0.5 x 3	1.68 x 1.09 x 5
**Reconstruction pixel size (mm x mm x mm)**	1.6 x 1.6 x 3.5	1.6 x 1.6 x 3.5	0.5 x 0.5 x 3	1.1 x 1.1 x 3
**Number of slices**	20	20	35/35/35	30
**Interslice gap (mm)**	0	0	0/0/0	-
**b-values (s/mm^2)/number of excitations**	100/2, 600/7, 1000/12	100/4, 1200/12	-	-
**Number of excitations**	-	-	3	1
**Fat saturation**	SPAIR	SPAIR	-	Spectral
**Parallel imaging (acceleration factor)**	SENSE (2)	SENSE (2)	SENSE (2)	SENSE (2)
**Acquisition time (min)**	4.4	3.4	5.1/5.1/5.2	3.25

* Used to build the apparent diffusion coefficient map by fitting signal intensity versus the b-values up to 1000 s/mm^2.

** Used to provide the maximum b-value images (b = 1400 s/mm^2 , obtained by interpolation).

Supplementary Table 2 - Acquisition parameters for 3.0 Tesla MRI of the prostate. DCE = dynamic contrast-enhanced; DWI = diffusion-weighted imaging; FOV = field of view; SENSE = sensitivity encoding; SPAIR = spectral adiabatic inversion recovery; TE = time of echo; TR = time of repetition; TSE = turbo spin echo; SS-EPI = single-shot echo-planar imaging; THRIVE = T1-weighted high-resolution isotropic volume examination; EPI = echo planar imaging.

	**Double sequence DWI**		**TSE T2-weighted imaging**	**DCE**
	**First sequence***	**Second sequence****		
**Sequence**	SS-EPI	SS-EPI	TSE	THRIVE
**Weighting**	DWI	DWI	T2	T1
**Acquisition**	axial	axial	axial/coronal/sagittal (at least 2 planes)	axial
**TR (ms)**	5350	5424	4727/3076/3714	3.5
**TE (ms)**	68	78	80/80/80	1.77
**Echo train lenght**	-	-	8/16/16	-
**EPI factor**	109	109	-	-
**Hald scan factor**	0.62	0.62	No	No
**FOV (mm x mm)**	200x200	200x200	180x180	200x200
**Acquisition voxel size (mm x mm x mm)**	2x2x3	2x2x3	0.6x0.6x3	1.2x1.2x8
**Reconstruction pixel size (mm x mm x mm)**	1.4x1.4x3	1.4x1.4x3	0.45x0.45x3	0.63x0.6x4
**Number of slices**	24	24	24/20/20	20
**Interslice gap (mm)**	0	0	0/0/0	0
**b-values (s/mm^2)**	100/1, 500/1, 1000/2	100/1, 2000/3	-	-
**Number of excitations**	-	-	2/1/1	1
**Fat saturation**	SPAIR	SPAIR	-	Spectral
**Parallel imaging (accelleration factor)**	SENSE (2)	SENSE (2)	SENSE (2.5/1/1)	SENSE (2)
**Acquisition time (min)**	3.2	3.2	5.3/4.2/5.1	6.16

* Used to build the apparent diffusion coefficient map by fitting signal intensity versus the b-values up to 1000 s/mm^2.

** Used to provide the maximum b-value images (b = 2000 s/mm^2).

Supplementary Table 3 – Details on the eleven index lesions discordantly categorized by reader 1 and reader 2 using the Prostate imaging reporting and data system (PI-RADS version 2.1).

PI-RADS categorization by Reader 1	PI-RADS categorization by Reader 2	Total discordant index lesions per PI-RADS category
2	4	5
3	4	2
4	2	2
4	3	2

Supplementary Table 4 – Details on combined PI-RADS-Likert categorization of the index lesions made by reader 1 and reader 2. ASAP = Atypical small acinar proliferation; DRE = digital rectal examination; DWI = diffusion-weighted imaging; ISUP = International Society of Urological Pathology; MRI = Magnetic Resonance Imaging; PI-RADS = Prostate imaging reporting and data system v2.1; PZ = peripheral zone; PZa = anterior peripheral zone; PSA = prostate specific antigen; PSAD = PSA-Density; R1 = reader 1; R2 =reader 2; T2WI = T2 weighted imaging; TZ = transitional zone.

	Number of lesions	PI-RADS category	Likert score	Prostate zone	Reason for adjusting with the Likert score	Biopsy
**R1** (n=28)	1	1	3	PZ	Contrast enhancement focus without a correlate on DWI, in association with diffuse signal heterogeneity on T2WI and PSAD > 0.15 ng/mL/mL	Atrophy/ subatrophy/ chronic inflammation
	1	2	3	PZ	Contrast enhancement focus without a correlate on DWI or T2WI, associated with PSAD = 0.21 ng/mL/mL and positive DRE	Chronic inflammation
	5	3	2	▪ PZ (n=3)<br>▪ TZ (n=2)	Low clinical suspicion of cancer, i.e. PSAD <0.15 ng/mL/mL, negative DRE, no familiy history	Active inflammation/ chronic inflammation
	2	4	2	▪ PZ (n=1)<br>▪ TZ (n=1)		Active inflammation/ chronic inflammation
	8	1	2	PZ		Atrophy/ subatrophy/ chronic inflammation
	2	2	2	PZ		Atrophy/ subatrophy/ chronic inflammation (one case) and ISUP 2 cancer (one case)
	3	4	3	PZ	▪ Case 1: PSAD 0.13 ng/mL/mL, history of long-standing increase in PSA value and repeated previous episodes prostatitis, negative DRE, no family history<br>▪ Case 2: PSAD 0.07 ng/mL/mL, negative DRE<br>▪ Case 3: PSAD 0.18 ng/mL/mL, history of long-standing increase in PSA value and previous repeated episodes of prostatitis, negative DRE	▪ Case 1: ISUP 4 cancer<br>▪ Case 2 and 3: ISUP 2 cancer
	4	4	4	▪ 3 PZ (n=3)<br>▪ TZ (n=1)	▪ Case 1: PZa: despite negative DRE, PSAD 0.32 ng/mL/mL and history of rapid increase in PSA value<br>▪ Case 2: positive DRE reasonably corresponding to the imaging finding<br>▪ Case 3: PSAD 0.24 ng/mL/mL and positive DRE<br>▪ Case 4 (in the TZ): PSAD 0.19 ng/mL/mL	▪ Case 1-3: ISUP 2 cancer<br>▪ Case 4: active inflammation and chronic inflammation

	2	2 upgraded to 3	4	TZ	▪ Case 1: rapid increase in the PSA level up to 4.91 ng/ml, markedly restricted diffusion<br>▪ Case 2: PSAD 0.19 ng/mL/mL, previous negative biopsy showing ASAP in the MRI suspicious region, markedly restricted diffusion	▪ Case 1: ISUP 2 cancer<br>▪ Case 2: Atrophy/ subatrophy/ chronic inflammation
**R2** (n=18)	1	2	3	PZ	PSAD 0.21 ng/mL/mL	Atrophy/ subatrophy/ chronic inflammation
	2	3	2	▪ TZ (n=1)<br>▪ PZ (n=1)	▪ Case 1 (TZ): PSAD levels 0.09 ng/mL/mL<br>▪ Case 2 (PZ): PSAD 0.08 ng/mL/mL	Atrophy, subatrophy, chronic inflammation
	4	4	2	PZ	▪ Case 1: fluctuating PSA, negative DRE, PSAD 0.04 ng/mL/Ml<br>▪ Case 2: PSAD 0.05 ng/mL/mL<br>▪ Case 3: PSAD 0.09 ng/mL/mL, previous prostatitis and decreasing PSA value, no familiy history<br>▪ Case 4: PSAD 0.14 ng/mL/Ml, negative DRE, no familiy history	▪ Case 1: ISUP 4<br>▪ Case 2-4: Active inflammation and chronic inflammation
	2	3	4	▪ PZ (n=1)<br>▪ TZ (n=1)	▪ Case 1 (PZ): PSAD 0.37 ng/mL/mL<br>▪ Case 2 (TZ): PSAD 0.21 ng/mL/mL	Atrophy, subatrophy, chronic inflammation
	6	4	3	PZ	▪ Case 1: PSAD 0.12 ng/mL/mL, negative DRE<br>▪ Case 2: PSAD 0.12 ng/mL/mL, negative DRE<br>▪ Case 3: PSAD 0.06 ng/mL/mL, negative DRE<br>▪ Case 4: PSAD 0.11 ng/mL/mL, negative DRE<br>▪ Case 5: PSAD 0.05 ng/mL/mL, negative DRE<br>▪ Case 6: PSAD 0.09 nf/mL/mL, negative DRE	▪ Case 1: ISUP 4 cancer<br>▪ Case 2-6: Atrophy/ subatrophy/ chronic inflammation
	3	4	5	PZ	▪ Case 1: PSAD 0.30 ng/mL/mL, positive DRE<br>▪ Case 2: PSAD level 0.34 ng/mL/mL, positive DRE<br>▪ Case 3: PSAD 0.17 ng/mL/mL, negative DRE	▪ Case 1: ISUP 5 cancer<br>▪ Case 2: ISUP 3 cancer<br>▪ Case 3: Active inflammation/ chronic inflammation
